# Exploring scenarios for implementing fast quantitative MRI

**DOI:** 10.1016/j.ejro.2025.100658

**Published:** 2025-05-08

**Authors:** Susan V. van Hees, Martin B. Schilder, Alexandra Keyser, Alessandro Sbrizzi, Jordi P.D. Kleinloog, Wouter P.C. Boon

**Affiliations:** a Copernicus Institute of Sustainable Development, Innovation Studies Section, Utrecht University, the Netherlands; b Computational Imaging Group for MR Diagnostics & Therapy, Center for Image Sciences, University Medical Center Utrecht, Utrecht, the Netherlands

**Keywords:** Implementation, Fast quantitative MRI, Relaxometry, Workflows, Qualitative research

## Abstract

**Purpose:**

MRI waitlists and discomfort from long scanning sessions are significant problems in clinical radiology. Novel multiparametric quantitative MRI techniques (qMRI) for radiological imaging enable acquisition of full-brain data within minutes to address these problems. While technical and clinical work is advancing, there has been limited research on implementing fast qMRI. This paper aims to identify implementation factors and scenarios within a healthcare setting facing rising demand, staff shortages, and limited capacity of MRI systems.

**Methods:**

The paper reports on data collected using qualitative methods: 1) Interviews and guided discussions, 2) co-creation workshop. Both steps involved key representatives with various backgrounds and expertise, such as radiologists, lab technicians, insurers, and patients.

**Results:**

Workshop participants visualised current and future workflows, which helped articulate implementation factors for qMRI. Supply and demand in MRI will change with increased accessibility and shortened timeslots. Three implementation scenarios came forward: 1) stable deployment, 2) extension to conducting more complex diagnostic exams, and 3) (more) preventive screening.

**Discussion and conclusions:**

This paper demonstrates challenges, solutions, and opportunities for successfully implementing fast qMRI in the clinic, and five lessons for adoption in the clinic: 1) importance of balancing perfectionism with confidence when it comes to clinicians’ expectations, 2) good use of Artificial Intelligence, 3) considering a learning curve associated with implementation, 4) regarding competing technologies, and 5) including patients’ experiences. Future research should investigate salient issues regarding future of AI in radiology and for moving imaging practices out of the clinic.

## 1 Introduction: fast quantitative MRI

Extended waitlists and patient discomfort from prolonged scanning sessions are major problems in Magnetic Resonance Imaging (MRI). Multiple contrast-weighted scans (or sequences) must be acquired for the radiological assessment of disease using MRI. Due to the sequential nature of this acquisition, standard MRI protocols are lengthy. Alternative novel multi-parametric quantitative MRI (qMRI) techniques, such as MR Fingerprinting (MRF) [1], synthetic MRI [2], STrategically Acquired Gradient Echo, [3] MultiPathway MultiEcho [4], Quantitative Transient Imaging [5] and Magnetic Resonance Spin TomogrAphy in Time-domain (MR-STAT) [6], are considered as fruitful strategies when aiming to shorten these protocols. These techniques acquire full-brain data within a few minutes. The primary output of a multiparametric sequence, as opposed to a conventional acquisition, is a set of quantitative parameter maps.

Conventional contrast-weighted images can be synthesised from the quantitative parameter maps utilising established physical signal models [7] or deep-learning-based approaches [8], [9], [10], [11], [12]. Therefore, all data necessary for the radiological inspection is acquired during the short multiparametric protocol, obviating the problem of long scan sessions. Recent literature shows synthetically generated contrast-weighted images have similar image quality compared to conventionally acquired contrast-weighted images [13], [14]. This advocates for increased adoption of fast, multiparametric MRI.

The adoption of multiparametric techniques could also overcome the major limitation of producing non-standardised image values associated with standard MRI protocols, providing opportunities to leverage these values for machine learning algorithms that aid in, e.g., automatic white matter anomaly detection or glioblastoma infiltration prediction [15], [16]. The obtained pixel intensity used to be based on arbitrary signal values that heavily depend on sequence-specific parameters. Minor alterations in sequence parameters can strongly influence signal values and subsequently impact the machine learning inference step [13], [14]. Multiparametric MRI offers information extracted from the data regarding physical, objective tissue properties. Potentially, this facilitates the applications of data-driven methods (machine learning), which rely on harmonised datasets for training. An example comes from Springer et al., who used the quantitative maps derived from an MRF protocol to distinguish between distinct types of gliomas [17], demonstrating the added clinical value of fast, quantitative MRI (qMRI).

While technical and clinical work is advancing fast qMRI, there has been limited research on its implementation and further development. Previous studies exploring the implementation of fast qMRI focused on a need for standardisation [18], enabling improved clinical decision-making through repeatable and accurate processes [19]. Moreover, radiologists are shown to play an important role in implementation processes and in adjusting workflows [20], [21]. The work presented here aims to complement such studies, broadening the range of implementation factors, covering impacts on workflows, and considering the healthcare context facing rising demand, personnel shortages, and the limited capacity of MRI systems [19], [22], [23], as well as implications for patients [24]. In a prospective, qualitative study, drawing on an established technology assessment approach (‘Responsible Research and Innovation’; see Appendix 1), we investigated the future impact of fast qMRI and explored possible solutions and opportunities for expected challenges. Stakeholders co-created plausible implementation scenarios for qMRI in Dutch clinical settings.

## 2 Methods

**Fig. 1:**
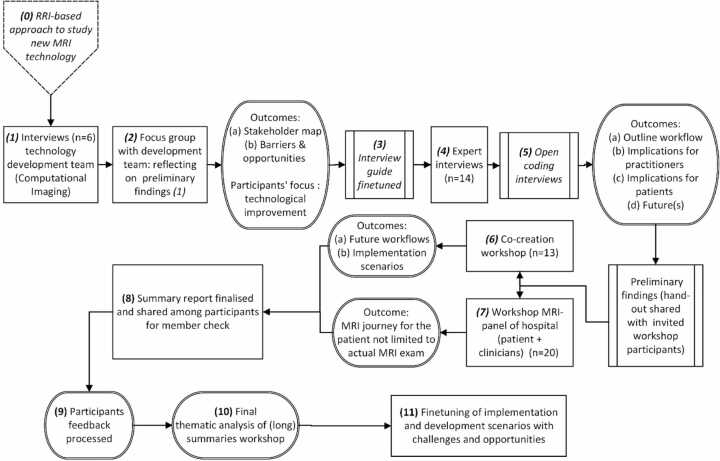
Overview of data collection steps.

We conducted an inductive study to explore implementation factors and scenarios of fast qMRI based on input from interviewees, focus groups, and workshop participants who represented relevant stakeholders of MRI. We applied a recognised sequence of qualitative methods, starting with interviews and guided discussions (focus groups), which fed into two co-creation workshops (Fig. 1). Our research covered the period from December 2021 to November 2022. We focused on one country – the Netherlands – because this allowed us to include the most extensive variety of stakeholders within one institutional context. The research protocol was assessed and exempted from medical ethical review by the medical ethics board of UMC Utrecht.^1^ All participants in the study gave written informed consent.

Technology developers working on a qMRI technology – specifically on MR-STAT – were interviewed (*n = 6;* see Appendix 2 for an overview of all participants; Step 1 in Fig. 1). The interviews were conducted online and took 45–60 min. They had a semi-structured format, covering opportunities and expected barriers associated with the technology and potential impacts on (clinical) workflows. Transcripts of the interviews were analysed in-depth, starting with open coding principles. Outcomes were used to formulate additional questions for a focus group with the technology developers (Step 2). Resulting insights into technical barriers fed into an enriched, finetuned interview guide that included potential non-technical implementation factors.

A second series of in-depth interviews was conducted with a broad range of participants, representing radiologists, lab technicians, patient representatives, healthcare insurers, vendors, policymakers, and AI experts (*n = 14*) (Steps 3 and 4). All participants were purposively selected using a stakeholder map drafted by the technology developers to complement the selection of the research team, aiming to include and represent a wide variety of stakeholders. We also asked interviewees for additional references. These semi-structured, online expert interviews were recorded, transcribed, and lasted 45–60 min. Two researchers independently coded the interview transcripts and discussed (dis)similarities to come to common themes in the material for further iteration to ensure intercoder reliability (Step 5). Examples of themes coming forward of this analysis include identified challenges such as methods to keep the profession of the radiologist sufficiently interesting for current radiologists despite the higher level of standardisation of protocols or communication tactics regarding shorter scanning times (e.g., will the quality be maintained). The identified themes served as input for two co-creation workshops. The first workshop (Step 6) covered the perspectives of various participants (*n = 13)* representing a wide range of stakeholder groups similar to the ones included in the interviews. They participated in the workshop in parallel break-out group discussions, reflecting on 1) current MRI workflows and implications for future workflows due to introducing fast qMRI, 2) expected challenges related to fast qMRI and possible solutions, and 3) prospective implementation scenarios. In a second workshop (Step 7), the same topics were discussed with a panel representing end-users (*n = 20*), including multiple patient representatives, nurses, medical specialists, radiation therapists, and radiologists. A presentation on MR-STAT, the main findings from the interviews, and the first workshop inspired the discussion. After analysing the recordings and transcripts of the workshops, a summary report was written (Step 8) and shared with workshop respondents and participants for a final validation check to confirm accuracy and authenticity. After integrating minor feedback (Step 9), a final round of analysis of extended summaries was done (Step 10), resulting in the implementation scenarios (Step 11).

## 3 Results

The current MRI workflows, the impact of fast qMRI on future workflows, anticipated implementation challenges, possible opportunities and solutions, and prospective implementation scenarios are presented below.

### 3.1 Current MRI and future fast qMRI workflows

**Fig. 2:**
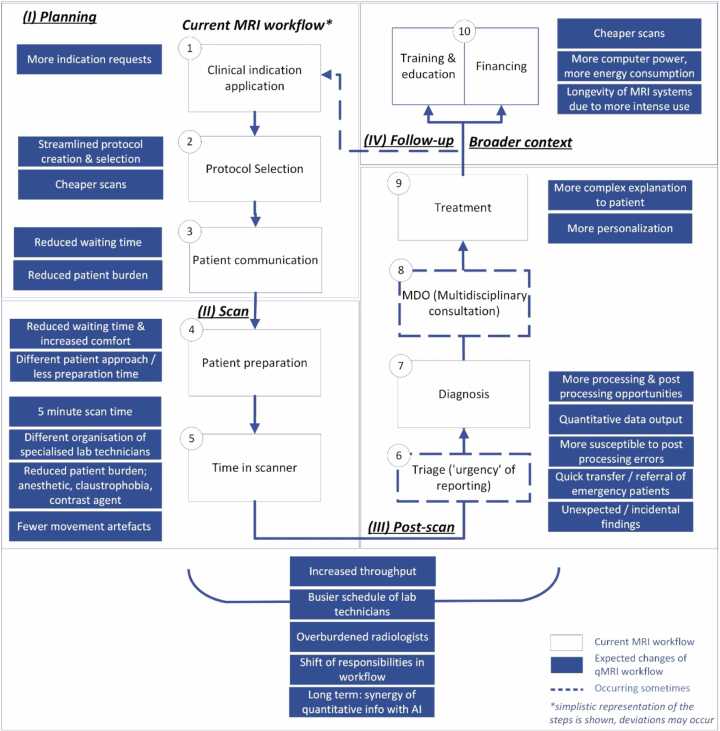
Visualization of the MRI workflow and implications of fast qMRI on workflows, drawing on the figure developed by Streit et al. [25] and developed with input from participants during workshops and interviews.

The white boxes in Fig. 2 visualise the current MRI workflow based on Streit et al. [25], further developed in the first co-creation workshop. The blue boxes indicate the expected implications of the implementation of fast qMRI.

As workshop participants and interviewees emphasised, the most notable implications concern changes in the supply of and demand for MRI. In the planning phase (I), research participants expect improved accessibility of an MRI exam. Due to shorter scan times, more exams are likely possible. The increased capacity might lead to lower thresholds for using MRI as part of clinical diagnosis and substituting scans currently performed with other imaging techniques (e.g., a Computed tomography (CT)-scan). Furthermore, requests for additional and continuous checks and more complex diagnostic scanning are expected.

In the scan phase (II), a projected increase in MRI requests will create a new type of capacity problem, as it would place a higher physical burden on lab technicians. Patients spending less time in the scanner allows more patients to be scanned daily, requiring more patients to be prepared on a given day. The post-scan phase (III) increase will also be felt as radiologists’ workload in evaluating images grows. The quantitative nature of MRI also creates post-processing opportunities. Scan data can be more easily used in AI applications, probably contributing to faster and more accurate diagnoses. More accurate scans are expected to create more opportunities for personalisation of treatment in the follow-up phase (IV). They help in 1) determining a priori the response of a tumour to therapy (i.e., radiosensitivity or chemosensitivity); 2) response evaluation of tumour on therapy (are quantitative values or the delta in quantitative values a predictor); 3) long-term follow-up of progressive neurodegenerative diseases. Yet more and faster scans may also result in longer waiting times to receive a diagnosis after the scan.

### 3.2 Expected challenges related to fast qMRI and possible solutions

Implications highlighted by the participants (Fig. 2) were discussed more elaborately in the workshops, as summarised in Table 1. Table 1 shows that although fast qMRI comes with promises, the implications also come with anticipated challenges and possible opportunities and solutions.

**Table 1:** Implications of implementing fast qMRI and associated challenges, opportunities, and solutions as proposed by workshop participants.

**Expected implications**		**Challenges**	**Possible opportunities and solutions**
I. Planning	More indication requests (applications for an MRI exam)	There is less flexibility in planning, and it is more complicated to ‘squeeze’ emergency scans.	Opportunities: − It is easier to schedule an exam with a different purpose than diagnostics (e.g., research and/or checks during treatment). − It is easier to ask for a first scan and additional or follow-up exams.
	Reduced waiting time before an exam (due to shorter time slots, shorter waiting lists, and time upfront) and reduced patient burden	Challenges highlighted under ‘II. Scan’.	Opportunity: − More patients per day due to shorter time slots per patient.
I. Scan	Shorter time slots needed per patient enable more scans per day, leading to: Tight(er) planning, i.e., more patients per hour	− The time a patient needs to be in the MRI room is reduced significantly. Preparation time remains, as well as some time afterwards. There is a risk that MRI machines are not optimally used if logistics is not thought through sufficiently. − Tighter planning implies a relative increase in time needed for patient preparation as part of the complete MRI process. − There is a higher chance of human errors in preparation for an exam as the number of patients to be prepared increases. − There is a higher number of patients who need help to get on and off the table, which intensifies the physical workload of lab technicians.	Solution: − While one patient is on the table during their 15-min slot, the next patient can already be prepared. − Opportunities: − Organise workflow with fewer specialists/highly trained professionals; e.g., shorter scan time affords radiologists more time for other tasks and creates an opportunity to task Physician Assistants with patient preparation. − Optimise time slots, considering efficiency and safety, as ensuring safety requires attention, so time slots must be wide enough for proper security checks during preparation.
	Reduced time in a scanner and increased comfort	− Patients warn that very short time slots may lead to distrust, as some connotate more time needed with more attentiveness and preciseness. They wonder if they can trust such fast techniques.	Solution: − Clear information about changes in the patient journey and before and after the MRI exam, i.e., good professional guidance during the entire journey, will contribute to the level of trust. Opportunities: − Higher comfort for the patient, as the time the patient is not supposed to move is reduced. − Reduced period of possible claustrophobic experience. − Fewer movement artefacts, leading to more correct scans.
	Longer-term expectation: Contrast agent is no longer needed	− Time needed for preparation decreases as preparation becomes less complex/non-invasive. Reduced complexity may bore out professionals currently tasked with this, leading to professionals leaving their jobs.	Opportunities: − Have assistants or nurses take over part of the workload of lab technicians. − Different approach to patients: less preparation time required and/or less complexity in preparation.
	Increased output: due to more scans, more processing is needed	− More scans lead to a higher workload for radiologists. − Potential delay in diagnosis due to increased workload for radiologists.	Solutions: (1) More radiologists are needed (but not considered realistic to fulfil the need completely). (2) A different work distribution/workflow organisation. E.g., by outsourcing reporting: a) to foreign countries (e.g., already done in the United Kingdom); b) to artificial intelligence (AI) to take over diagnosis (partly); c) or partly to different kinds of professionals (e.g., more tasks for nurse practitioners could lead to lower pressure on or other tasks for lab technicians; d) The number of ‘simple,’ easy-to-process scans is increasing, enabling the outsourcing of evaluation to other specialists. (3) Incidental findings need a follow-up, potential early detection and prevention, and risk of unnecessarily acting on findings. (4) Instead of more scans, use time for more complex diagnostic exams rather than on more ‘normal’ exams.
I. Post-scan	Output changes from qualitative to quantitative data. (At least) in the first stage, an image will be reconstructed synthetically to ease the transition to working with new images	− The use of AI in diagnosis, with the possibility of more incidental findings, implies a need for more, and more elaborate scans. − Examining a scan during the MRI exam is no longer possible.	Opportunities: − AI opens up more opportunities for personalised treatment. Examples are the analysis of more extensive data sets to compare, enabling better prediction and tailoring treatment plans. − To substitute for quality control during the MRI exam, a quality check should be introduced to ensure that a scan was successful and anticipate potential errors.
	More post-processing is possible (as more data can be collected with one scan)	Further analysis requires additional work from radiologists.	Opportunities: − Outsourcing to foreign countries: national regulation needs to be considered. For example, registration in the Register for Professions in Individual Health Care is required in the Netherlands. − Outsourcing to other professionals: e.g., training physician assistants/ doctors’ assistants in reporting. − However, if less complicated images are no longer reported by radiologists but outsourced, the average complexity of each MRI image that needs to be assessed by radiologists increases. This can enrich work pleasure but may also create a higher mental burden, i.e., only seeing the ‘worst cases.’
	Impact on organisation and planning in the broader context that needs attention:		
Broader context	AI application and increased demand for MRI exams lead to an increase in: − *Energy demands*: energy consumption is expected to increase due to the more intense use of machines that can make more scans per day if time-slot per scan is shortened. This also leads to higher environmental impact. − *Finances*: Financing structures currently differ between universities and peripheral hospitals in the Netherlands. Changes in workflows and increased demand will impact agreements with insurers. − *Training and education:* starting in medical school and centring primarily on training in practice, training, and education are crucial for imaging quality. Attention to the entire ‘patient journey’ is essential in current and future workflows, emphasising patients’ interests in: a) Expectations of the exam and (if applicable) previous experiences, e.g., anxiety due to duration, claustrophobic feelings, and sounds; b) Place (timing) of the exam in the process, e.g., some patients experienced it as more difficult to wait for results when they know the exam had already taken place; c) Own role as a patient, for instance, in requesting/negotiating about an MRI over a CT; d) Environment/context: everything has meaning for patients, as getting an MRI exam is often emotionally loaded. Examples include variations in lighting within the room or on the equipment, changes in personnel during the exam – such as one person assisting the patient into the machine and another helping them out – along with differences in what people say or do not say, and their expressions or demeanour at the end of the exam.		

### 3.3 Implementation scenarios

Based on the implications, challenges, solutions, and opportunities of fast qMRI, the participants created several plausible future implementation scenarios, distinguishing shorter-term scenarios from more distant but still probable ones. The scenarios primarily relate to new time allocation in the clinic, as shorter exam times open up opportunities for reimagining the workflow:

• Scenario 1: The number of MRI exam requests will increase. Actual time in the scanner will be shorter, allowing the possibility of scheduling shorter slots for the same exam. More exams per hour imply that MRI exams have become much more accessible and a more reasonable substitute for other imaging technologies that are currently much cheaper and have lower waiting times, such as CT scans.
• Scenario 2: The existing number of exams remains stable or slightly increases. Time that comes available will be used to extend selected exams for more in-depth, complex diagnoses or to create space for clinically relevant scientific research, e.g., distinguishing actual progression from therapy-related effects. Longer exams with qMRI can be valuable, as they provide more data per hour available.
• Scenario 3: The use of MRI will be extended, including preventive screening next to diagnosis and treatment. Increased accessibility, leading to more room for screening, could contribute to the prevention and early detection of diseases. However, this scenario has its downsides, as it will come with more ‘incidental findings’ and false positives, as well as demands put on doctors.

When reflecting on the scenarios, participants remarked that next to changes in time allocation and changes in workflows, tasks, and responsibilities, fast qMRI potentially facilitates AI applications in imaging, in particular when it comes to diagnostics. Another more futuristic scenario involved the analysis of qMRI values in discerning between active and inactive multiple sclerosis lesions, potentially making gadolinium-based contrast agents redundant for MRI exams of this specific patient category [26]. This simplifies the preparation for an exam, as no injections are needed. In such a scenario, an increased extramuralisation of MRI exams is expected, with potentially more screenings, e.g., in private clinics and general practitioner offices.

For responsible implementation and development in the near future, a recurring pathway considered by participants is to start simple and in low-risk areas, like musculoskeletal applications, and to expand to other clinical indications incrementally. From an organisational perspective, planning all similar exams on the same (part of the) day allows for more efficient planning, though less flexible (i.e., regarding emergency exams and/or personnel issues).

## 4 Discussion and conclusions

In this paper, we explore the expected impact of the introduction of fast qMRI on imaging processes, as well as on the entire clinical workflow and the broader healthcare setting. Research participants reflected on current practices and anticipated future workflows involving qMRI, articulating challenges, opportunities, and solutions. Furthermore, they co-created scenarios that indicate plausible future applications of fast qMRI in the field of imaging and radiology in particular. These insights confirmed lessons from previous studies on how shorter exams are expected to benefit patients due to, for instance, fewer movement artefacts and higher comfort, but also lower costs [27], [28], [29].

From our study, five lessons can be distilled for the implementation and development of fast qMRI:

1) Clinicians should strike a balance between perfectionism and confidence. It may become tempting to request an MRI exam when there is increased availability, aiming for higher certainty or confirmation of diagnosis. However, participants also emphasised that unnecessary exams should be avoided.
2) Fast qMRI will not only change the workflow and ease access to MRI, but it also creates opportunities for the application of AI. For a meaningful exam and optimal use of AI’s potential, an even more precise formulation of the investigation request (‘indication’) is required since no alterations during the exam can be made as is currently sometimes done when during the exam something is noticed on the image for which more exact information or details are needed.
3) Space for experimentation and exploration is needed when implementing qMRI to enable learning from changes in the process and adopt these lessons in dealing with new types of data. This is necessary to optimise the use of the latest technique and to deal with expected ‘teething problems’ that are to be expected when implementing a new, more complex technology. A strategy proposed during the first workshop was to start ‘simple,’ with body parts that are less complex to scan, e.g., start with legs rather than brain scans.
4) Be aware of external, field-level developments impacting development and implementation opportunities, such as introducing other imaging technologies.
5) The patient’s experience will be impacted and needs to be considered. While the expectation is that their comfort will increase due to shorter times on the MRI table, clear and good communication about changes during the planning, preparation, and afterwards are considered essential for patients’ confidence in the clinical process and the MRI exam in particular. Patients value shorter pre-scan timelines but do not favour longer time-to-diagnosis in the post-scan phase, which might result from the higher workloads of radiologists. Moreover, when AI is introduced to automate parts of the process, it presumably impacts communication between specialists and patients and the decisions to be made about treatment.

Additional studies are needed to expand knowledge further and validate insights from this explorative and prospective, though small-scale study. Similar workshops in different countries and healthcare systems could enrich the transferability and translation of knowledge. Alternative study designs could increase the number and variety of stakeholders represented. Although mentioned, the focus on potential AI applications has not yet been fully explored. Therefore, future investigations should aim to gain additional insights into stakeholder perspectives on the future use of AI in healthcare, focusing on salient issues such as trustworthiness, ethics, and commercialisation [30], [31].

Reflections in these workshops on the value and possible implementation scenarios for fast qMRI open up further discussion on how to anticipate and design a plausible future in radiology. Not only are techniques changing, but there also needs to be further discussion on how tasks and responsibilities in the imaging workflow can be (re)divided to accommodate a sustainable and high-quality future in radiology. A direction to further explore could be the implications of moving imaging practices out of the clinic.

This study sought to identify challenges, solutions, and opportunities to successfully implement fast qMRI in the clinic. Balancing perfectionism with confidence regarding clinicians’ expectations, using artificial intelligence, and considering the learning curve associated with implementation, competing technologies, and patients’ experiences are essential to adoption in the clinical routine. Future research should investigate salient issues regarding the future of AI in radiology and moving imaging practices out of the clinic.

## CRediT authorship contribution statement

**Wouter P.C. Boon:** Writing – review & editing, Supervision, Methodology, Investigation, Funding acquisition, Formal analysis. **Jordi P.D. Kleinloog:** Writing – review & editing, Methodology, Investigation, Formal analysis, Data curation, Conceptualization. **Alessandro Sbrizzi:** Writing – review & editing, Supervision, Resources, Project administration, Investigation, Funding acquisition, Formal analysis. **Alexandra Keyser:** Writing – review & editing, Visualization. **Martin B. Schilder:** Writing – review & editing, Writing – original draft, Formal analysis. **Susan V. van Hees:** Writing – review & editing, Writing – original draft, Visualization, Methodology, Investigation, Formal analysis, Data curation, Conceptualization.

## Ethical statement

The research protocol was assessed and exempted from medical ethical review by the medical ethics board of UMC Utrecht. All participants in the study gave written and verbal informed consent. Declaration METC no. 22-475/DB, d.d. 1 March 2022.

## Funding

This publication is part of the project ‘Responsible implementation of quantitative MRI’ [with project number 18749 of the research programme HTSM MVI top-up which is financed by the Dutch Research Council (NWO)].

## Declaration of Competing Interest

The authors declare the following financial interests/personal relationships which may be considered as potential competing interests: Martin B Schilder reports a relationship with Philips that includes: funding grants (different project, which start later, on other topics). Jordi Kleinloog reports a relationship with Philips that includes: employment (started after he finalised his contribution to this project/the manuscript’s content). If there are other authors, they declare that they have no known competing financial interests or personal relationships that could have appeared to influence the work reported in this paper.

## Figures and Tables

**Table:** 

Overview of Research participants*			
	Type of expertise	Interview	Workshop 1
RM01	Radiologist, Peripheral Hospital	29–3–2022	
RM02	Director of Clinical Science MR at Tech company	31–3–2022	15–9–2022
RM03	Fund manager	31–3–2022	15–9–2022
RM04	MRI department manager, University Hospital	31–3–2022	
RM05	Computational Imaging Group Manager, University Hospital	6–4–2022	15–9–2022
RM06	Medical Advisor, Insurance Company	6–4–2022	
RM07	Chief Technology Officer MRI startup	13–4–2022	
RM08	Radiologist, University Hospital	7–4–2022	15–9–2022
RM09	Radiologist, University Hospital	14–4–2022	15–9–2022
RM10	Radiologist and Educational Coordinator, University Hospital	24–3–2022	
RM11	Entrepreneur in Medical technology	21–4–2022	
RM12	Neurologist, University Hospital	9–5–2022	
RM13	Patient (representative)	19–5–2022	
RM14	Division Manager, University Hospital	16–8–2022	
RM15	Funding Agency		15–9–2022
RM16	Radiologist		15–9–2022
RM17	N/a		
RM18	Patient (representative)		15–9–2022
RM19	Social scientist		15–9–2022
RM20	Knowledge transfer office		15–9–2022
RM21	Technical, medical specialist		15–9–2022
RM22	Radiologist, University Hospital		15–9–2022
RM23	Radiologist, University Hospital		15–9–2022
RM24	Lab technician (radiotherapy)		15–9–2022

* in addition to this list of interviewees and workshop 1 participants, 20 people participated in workshop 2, which took place on September 22, 2022, with the entire MRI patient panel of a university hospital, existing patients (representatives), and professionals (including a radiologist, neurologist, radiotherapist). No recording was made of that workshop. Extensive notes of the discussion with and between patients, patient representatives, and professionals were made and used for analysis.

## Appendix 1 Approaches to assessing the future application of technologies

Emerging technologies are associated with a dilemma: in the early stages of development, the impact of technology is not easy to predict, yet it can be guided. The impact becomes more apparent in an advanced stage, but the possibility of steering development diminishes [32]. To still be able to assess the future implications of emerging technologies, innovation studies scholars have introduced well-established theories and methods [33], [34].

Responsible Research and Innovation (RRI) is a prominent approach to technology assessment. RRI’s central premise is that by exploring potential ethical, legal, and societal issues, innovators can anticipate and even work on solutions to pre-empt these issues from happening, which might boost acceptance and implementation of the technology [33]. RRI emphasised four dimensions [35: p.1]: 1) anticipation: study the potential impacts of technology from an early stage and uncover expectations and ‘what-if’-scenarios; 2) reflexivity: instigate thinking amongst technology developers and users about what the technology should be about and what this means for their responsibilities; 3) inclusion: incorporate a wide range of stakeholders and disciplines – even those of the ‘unusual suspects’ – and try to integrate their perspectives; and 4) responsiveness: make sure that the preferences and values are incorporated into the design of the innovation. In the past decade, the RRI approach has been applied to health technologies and innovations [36].

## Footnotes

1 Declaration METC no. 22-475/DB, d.d. 1 March 2022.
